# Hydrogen protects lung from hypoxia/re-oxygenation injury by reducing hydroxyl radical production and inhibiting inflammatory responses

**DOI:** 10.1038/s41598-018-26335-2

**Published:** 2018-05-22

**Authors:** Meihong Chen, Jie Zhang, Yun Chen, Yan Qiu, Zi Luo, Sixia Zhao, Lei Du, Dongbo Tian

**Affiliations:** 1 Department of Respiratory Medicine, The Sixth Affiliated Hospital of Guangzhou Medical University, Qingyuan, Guangdong, 511500 China; 2 Department of Pathology Laboratory, West China Hospital, Sichuan University, Chengdu, Sichuan 610041 China; 3 Department of Anesthesiology and Translational Neuroscience Center, West China Hospital, Sichuan University, Chengdu, Sichuan 610041 China; 4 Department of Anesthesiology, Loudi Central Hospital, Loudi, Hunan 417000 China; 5 Department of Anesthesiology, Xiangtan Central Hospital, Xiangtan, Hunan 411100 China

## Abstract

Here we investigated whether hydrogen can protect the lung from chronic injury induced by hypoxia**/**re-oxygenation (H/R). We developed a mouse model in which H/R exposure triggered clinically typical lung injury, involving increased alveolar wall thickening, infiltration by neutrophils, consolidation, alveolar hemorrhage, increased levels of inflammatory factors and recruitment of M1 macrophages. All these processes were attenuated in the presence of H_2_. We found that H/R-induced injury in our mouse model was associated with production of hydroxyl radicals as well as increased levels of colony-stimulating factors and circulating leukocytes. H_2_ attenuated H/R-induced production of hydroxyl radicals, up-regulation of colony-stimulating factors, and recruitment of neutrophils and M1 macrophages to lung tissues. However, H_2_ did not substantially affect the H/R-induced increase in erythropoietin or pulmonary artery remodeling. Our results suggest that H_2_ ameliorates H/R-induced lung injury by inhibiting hydroxyl radical production and inflammation in lungs. It may also prevent colony-stimulating factors from mobilizing progenitors in response to H/R-induced injury.

## Introduction

Hypoxia/re-oxygenation (H/R) is common during acute exacerbation and remission of asthma, bronchiectasis and early chronic obstructive pulmonary disease (COPD), and it can result in pulmonary inflammatory response^1^ and aggravate existing lung disease. H/R increases the production of reactive oxygen species (ROS) by the mitochondrial electronic transport chain^1^, NADPH oxidase^2^ and xanthine oxidase^3^. Under physiological conditions, ROS are produced at low levels to act as signaling messengers to maintain cellular functions^4^. Excessive production of ROS, particularly hydroxyl radicals as a result of H/R, oxidatively damages nucleic acids, lipids and proteins. ROS also induces polymorphonuclear leukocytes adhering to the pulmonary microcirculation^5^ to damage pulmonary epithelial cells^6^, vascular smooth muscle cells^7^, vascular endothelial cells^8^, and alveolar epithelial type II cells^9^. ROS in lung can initiate inflammatory responses by activating redox-sensitive transcription factors, including activator protein-1, hypoxia-inducible factor-1 and nuclear factor-kappa B^10,11^, which up-regulate expression of granulocyte macrophage colony-stimulating factor (GM-CSF)^12^, granulocyte colony-stimulating factor (G-CSF)^13^ and erythropoietin (EPO)^14^. These three factors may help initiate lung injury and pulmonary vascular remodeling by promoting lung inflammation^15^, increasing proliferation of pulmonary vascular smooth muscle cells^16^. Besides, alveolar macrophage (AM) was reported as a critical promoter to orchestrate pulmonary H/R injury via up-regulation of proinflammatory factors^17,18^. In this way, H/R-induced pulmonary inflammation is characterized by the recruitment of macrophages and neutrophils and by up-regulation of proinflammatory factors such as tumor necrosis factor (TNF)-α, interleukin (IL)-β and IL-6^19,20^.

Hydrogen (H_2_) is a non-toxic, colorless, odorless and transparent gas that is easily reducible, so it can react directly with strong oxidants such as hydroxyl radical^21^. It exerts anti-inflammatory, anti-apoptotic, pro-metabolic effects, and it can alter gene expression patterns^22^. It has shown therapeutic effects in animal models of Parkinson disease^23^, type 2 diabetes^24^, myocardial infarction^25^, acute hypoxia-induced brain injury^26^ and pulmonary infection^27^.

These results raise the question of whether H_2_ can attenuate H/R-induced lung injury. The present study explored this question using a mouse model of chronic H/R lung injury.

## Results

### H_2_ inhalation attenuates H/R-induced lung injury

**Figure 1:**
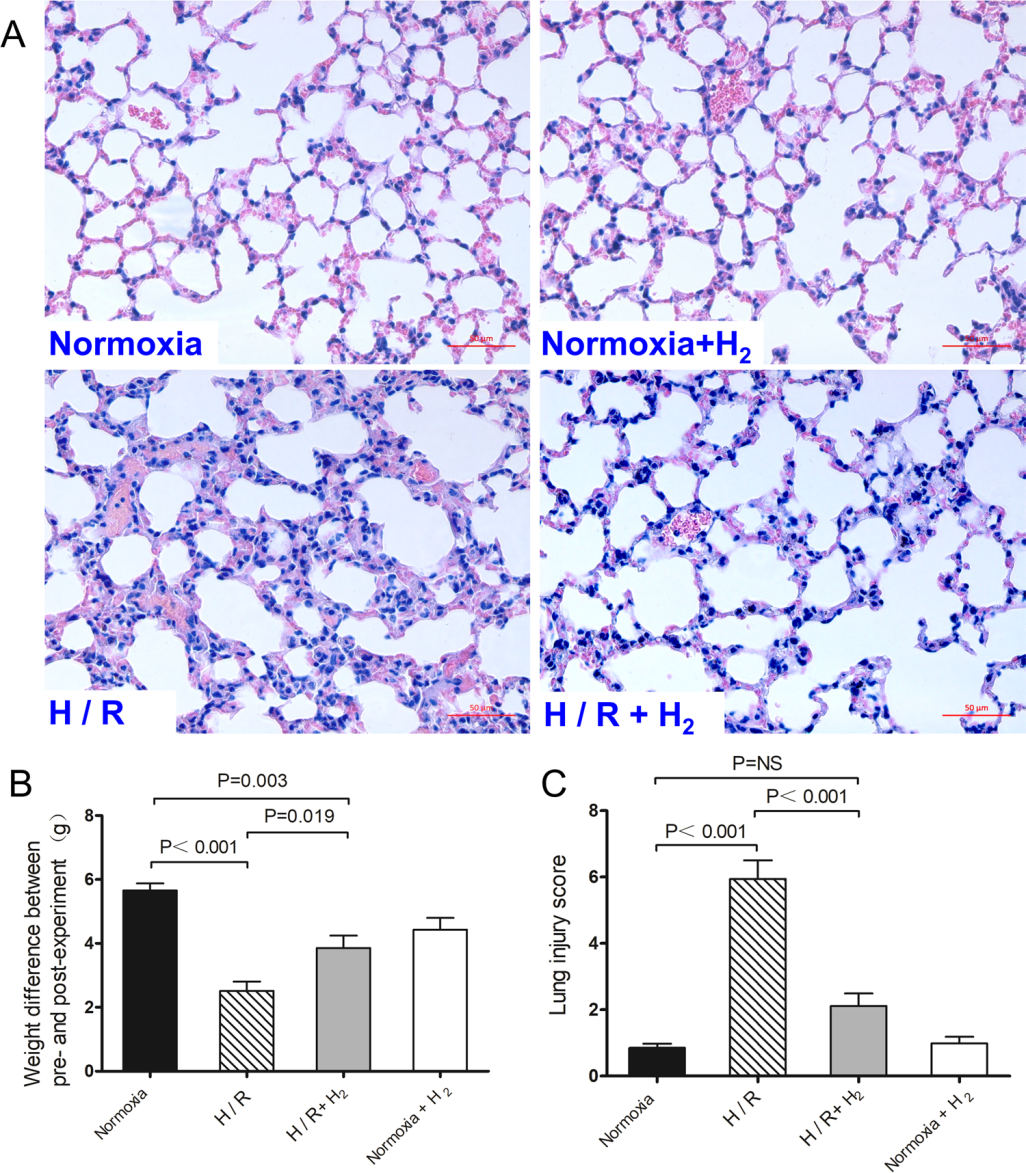
Hydrogen inhalation reduced H/R-induced lung injury. Mice were exposed for 4 weeks (8 h per day) to hypoxia, hypoxia with 4% H_2_ or normoxia with 4% H_2_ and then housed in normoxia (16 h per day). Control mice were housed in normoxia (n = 10 in each group). (**A**) Hematoxylin-eosin staining of lung tissues. (**B**) Change in weight between before and after the experiment. (**C**) Lung injury scores. Scale bar, 50 *μ*m. Data shown are mean ± SEM. ns, not significant.

To investigate the effects of H_2_ on H/R-induced lung injury, male C57BL/6 mice 8 weeks old were exposed for 4 weeks (8 h per day) to hypoxia (10% O_2_, 90% N_2_), hypoxia combined with H_2_ (10% O_2_, 4% H_2_, 86% N_2_) or normoxia combined with H_2_ (21%O_2_, 4%H_2_, 75%N_2_), and then housed in normoxia for the other 16 h to allow re-oxygenation. Control mice were continuously exposed to normoxia (21% O_2_, 79% N_2_) for 4 weeks. After 4 weeks of H/R, significant lung injury appeared that was characterized by alveolar wall thickening, infiltration of neutrophils into lung interstitium and the alveolar space, consolidation, and alveolar hemorrhage (Fig. 1A). In H/R-exposed mice, lung injury score was significantly higher than in normoxia-treated mice (5.94 ± 1.96 *vs*. 0.85 ± 0.13, p < 0.001; Fig. 1C), and H/R-exposed animals experienced a smaller increase in body weight (5.65 ± 0.22 *vs*. 2.52 ± 0.30 g, p < 0.001; Fig. 1B). H_2_ significantly attenuated H/R-induced infiltration of inflammatory cells and alveolar wall thickening, and it significantly decreased lung injury score (2.11 ± 0.38, p < 0.001 *vs*. H/R). H_2_ also led to a larger increase in body weight in hypoxic mice (3.86 ± 0.39 g, p = 0.019 *vs*. H/R), although the weight of hypoxic mice was still lower than that of normoxia-treated mice (p = 0.003).

**Figure 2:**
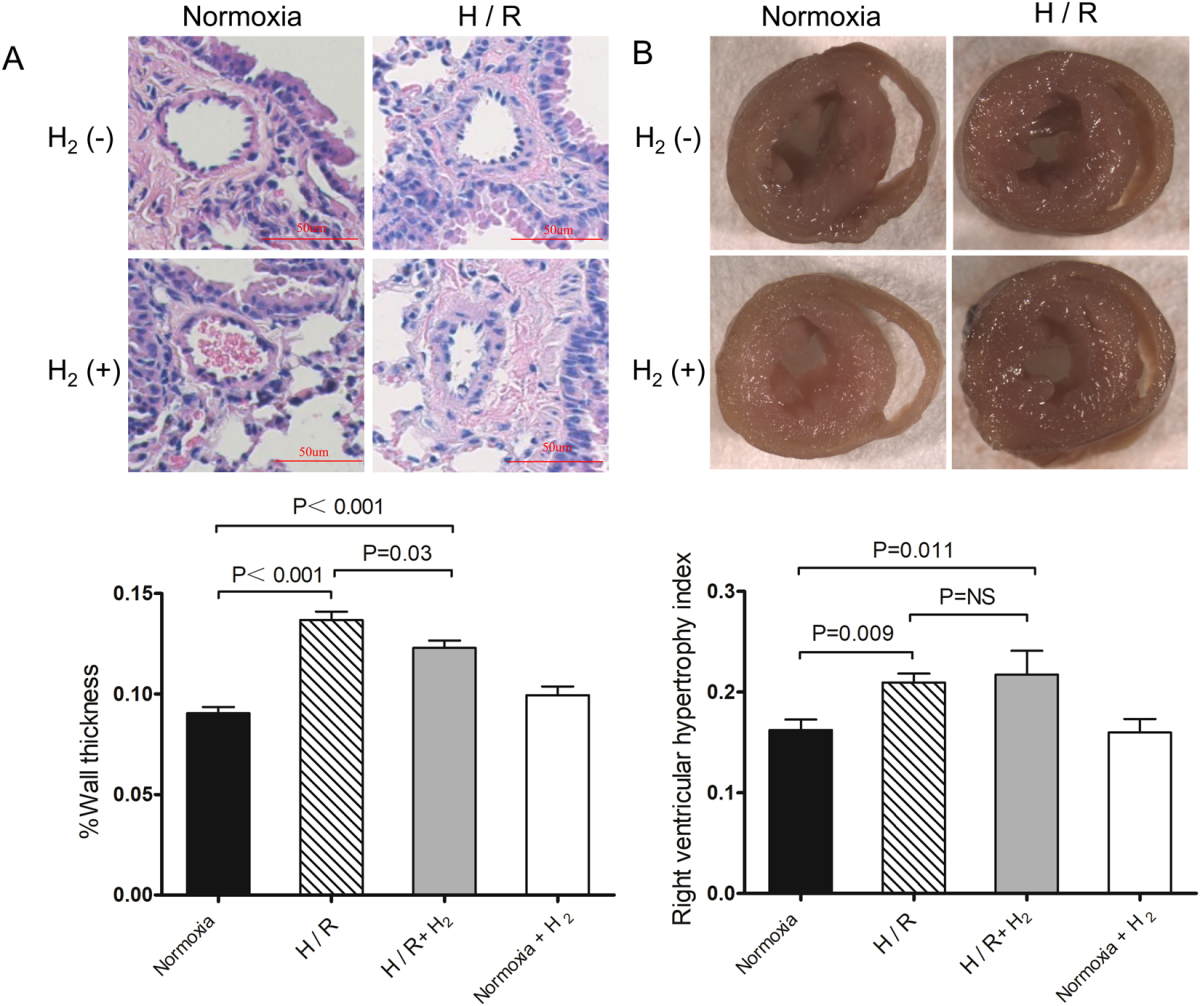
Limited effect of hydrogen on H/R-induced pulmonary artery remolding. Mice were treated as described in Fig. 1 (n = 10 in each group). (**A**) Representative sections stained with hematoxylin-eosin (top) and percentage of wall thickness of pulmonary arterioles in the lungs (bottom). (**B**) Mouse hearts in cross section (top) and right ventricular hypertrophy index (see Methods) (bottom). Data shown are mean ± SEM. ns, not significant.

To evaluate the effect of H_2_ on pulmonary artery remodeling, we measured the percentage of medial wall thickening and right ventricular hypertrophy proliferative index (RHVI). The percentage of medial wall thickening significantly increased after 4 weeks of H/R (0.137 ± 0.004 *vs*. 0.090 ± 0.003 *μ*m, p < 0.01; Fig. 2A), as did RHVI (0.209 ± 0.009 *vs*. 0.162 ± 0.010 g, p < 0.01; Fig. 2B). H_2_ slightly reduced H/R-induced medial wall thickening (0.123 ± 0.004 *μ*m, p = 0.03 *vs*. H/R), but not H/R-induced RHVI (0.217 ± 0.023 g, p = 0.69 *vs*. H/R).

These results suggest that H_2_ attenuates lung injury, although it does not substantially affect pulmonary artery remodeling induced by H/R.

### H_2_ inhalation reduces H/R-induced production of hydroxyl radicals

**Figure 3:**
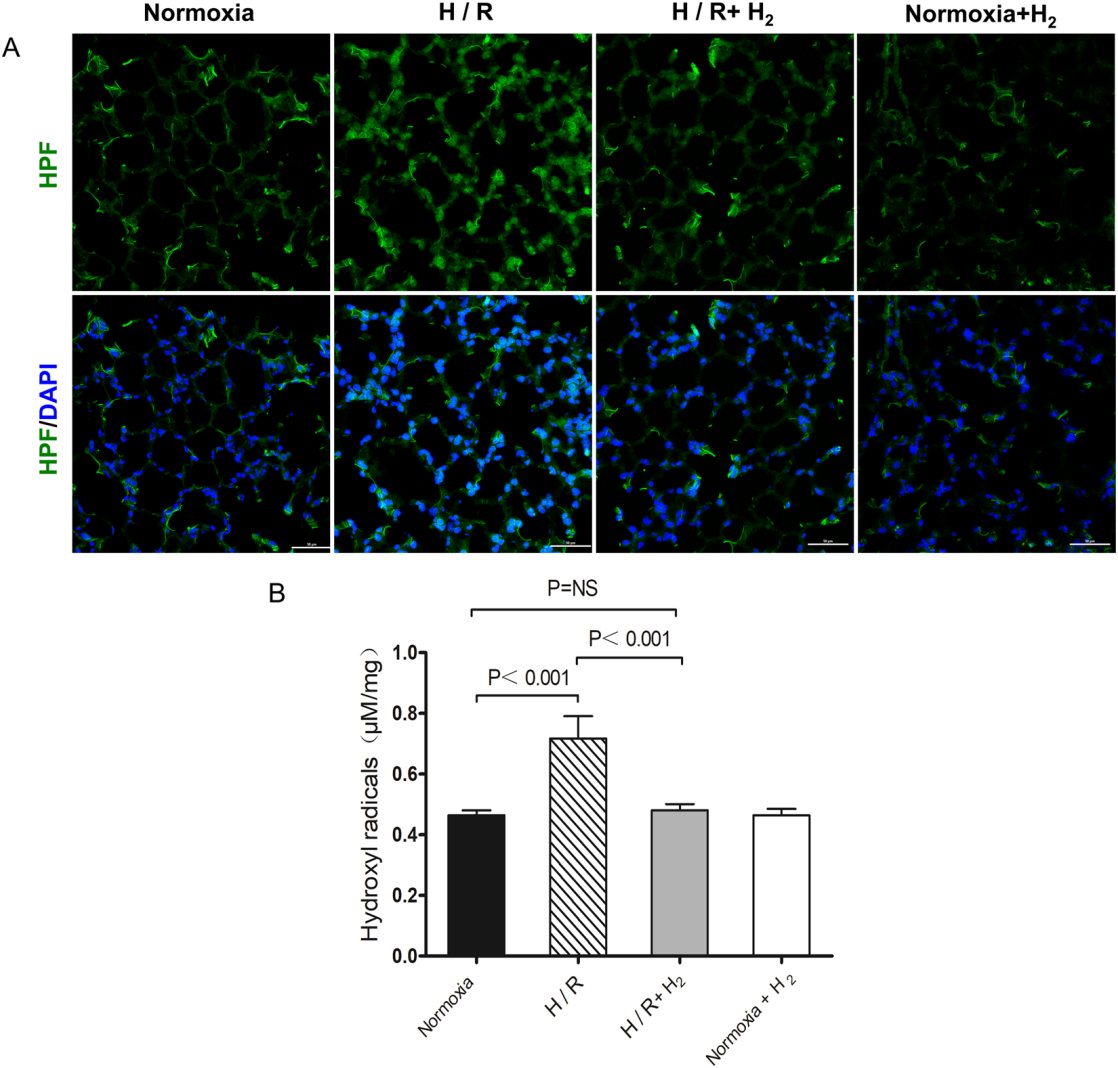
Inhibition of H/R-induced production of hydroxyl radicals by hydrogen. Mice were treated as described in Fig. 1 (n = 6 in each group). Lung samples were harvested, stained for hydroxyl radicals and quantitated. (**A**) Representative staining of lung tissue for hydroxyl radicals (original magnification, ×40). Sections were stained with hydroxyphenyl fluorescein solution and DAPI. Scale bar, 50 *μ*m. (**B**) Quantitation of hydroxyl radicals in lung tissue. Data shown are mean ± SEM. ns, not significant.

To begin to understand how H_2_ inhalation can protect lung from H/R-induced injury, tissue from exposed animals was sectioned and stained for hydroxyl radicals (Fig. 3A), levels of which were then quantified by chromatometry (Fig. 3B). H/R triggered a significant increase in hydroxyl radicals in lung tissue (0.72 ± 0.07 *vs*. 0.46 ± 0.02 *μ*M/mg, p < 0.001 *vs*. normoxia), which was markedly lower in the presence of H_2_ (0.48 ± 0.02 *μ*M/mg, p < 0.001 *vs*. H/R, p = 0.79 *vs*. normoxia).

### H_2_ inhalation attenuates both pulmonary and systemic inflammatory response

**Figure 4:**
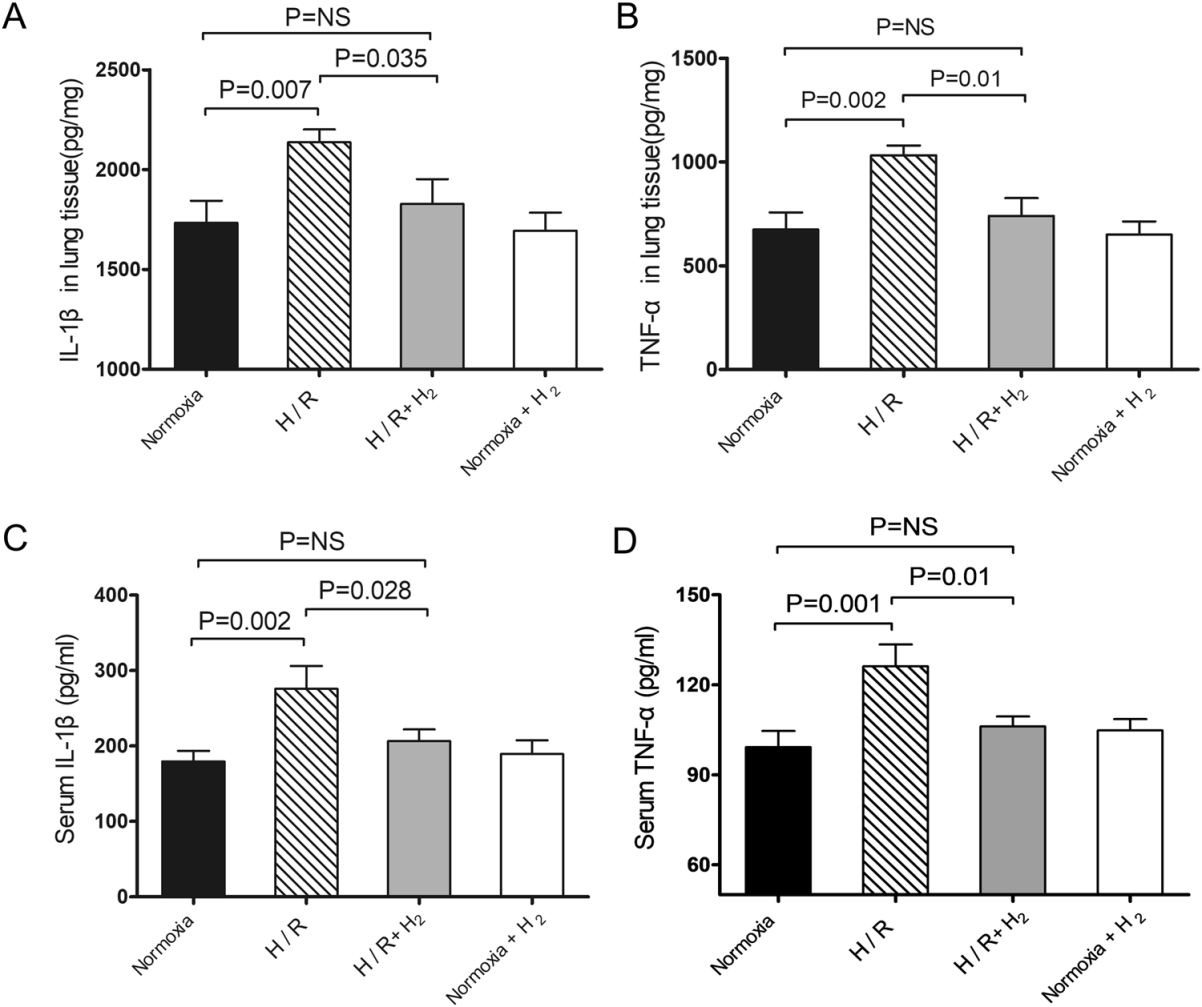
Down-regulation of pulmonary and systemic inflammatory responses by hydrogen. Animals were treated as described in Fig. 1 (n = 10 in each group). The right lung was harvested and homogenized at the end of experiments. After centrifugation, supernatants were harvested and assayed for IL-1β (**A**) and TNF-α (**B**) using commercial ELISA kits. Blood samples were also extracted and centrifuged for assaying IL-1β (**C**) and TNF-α (**D**) in serum. Data shown are mean ± SEM. ns, not significant.

To verify the effect of H_2_ on lung and systemic inflammation induced by chronic H/R, inflammatory factors were assayed in lung tissue and serum. Lung tissue from H/R-exposed mice showed significantly higher levels of IL-1β and TNF-α than tissue from normoxia-exposed mice (IL-1β: 2137 ± 63 *vs*. 1734 ± 110 pg/mg; TNF-α: 1032 ± 48 *vs*. 675 ± 82 pg/mg; both p < 0.01 *vs*. H/R; Fig. 4A,B). Similar results were observed in serum (IL-1β: 276 ± 31 vs. 180 ± 14 pg/ml; TNF-α: 126 ± 7 vs. 99 ± 5 pg/ml; both p < 0.01 *vs*. hypoxia; Fig. 4C,D). H/R in the presence of H_2_ led to levels of IL-1β in lung tissue that were similar to those in normoxia-treated animals (1828 ± 124 pg/mg, p < 0.05 *vs*. H/R, p = ns *vs*. normoxia). Similar results were observed for levels of IL-1β in serum (206 ± 16 pg/ml), levels of TNF-α in lung tissue (740 ± 85 pg/ml) and levels of TNF-α in serum (106 ± 3 pg/ml) (all p < 0.05 *vs*. H/R, p = ns *vs*. normoxia; Fig. 4A–D).

**Figure 5:**
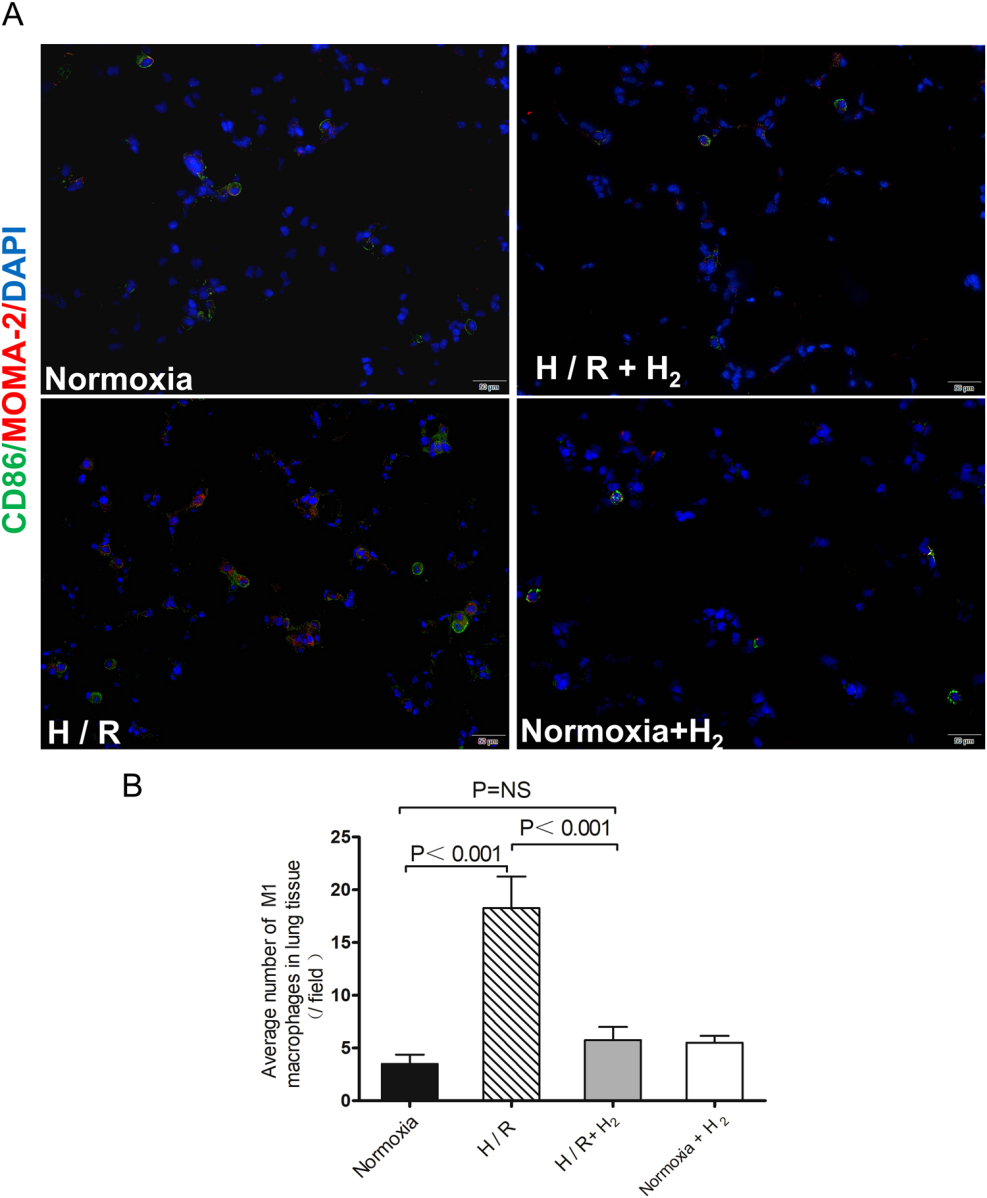
Staining (**A**) and quantitation (**B**) of M1 macrophages in the left lung. Animals were treated as described in Fig. 1 (n = 4 in each group). M1 macrophages were determined by immunofluorescence double staining with monocyte/macrophage marker (MOMA-2) and CD86. Sections were examined by confocal microscopy. The number of M1 macrophages in lung tissue was counted in one 200× field for each animal. Scale bar, 50 *μ*m. Data shown are mean ± SEM. ns, not significant.

Since the primary source of inflammatory factors in the lung is thought to be pulmonary M1 macrophages, lung tissue from H/R-exposed animals was stained for M1 macrophages. These cells were abundant in lung tissue from animals exposed only to H/R, but they were rare in tissue from animals exposed to H/R in the presence of H_2_ and in tissue from normoxia-treated animals (Fig. 5A,B).

Our observation of increased levels of inflammatory factors, leukocytes, neutrophils and GM-CSF in circulation as a result of H/R suggests that this exposure induces a systematic inflammatory response, consistent with previous work^28–30^. Our results further suggest that H_2_ attenuates this response in mice.

### H_2_ inhibits up-regulation of G-CSF and GM-CSF without affecting EPO

**Figure 6:**
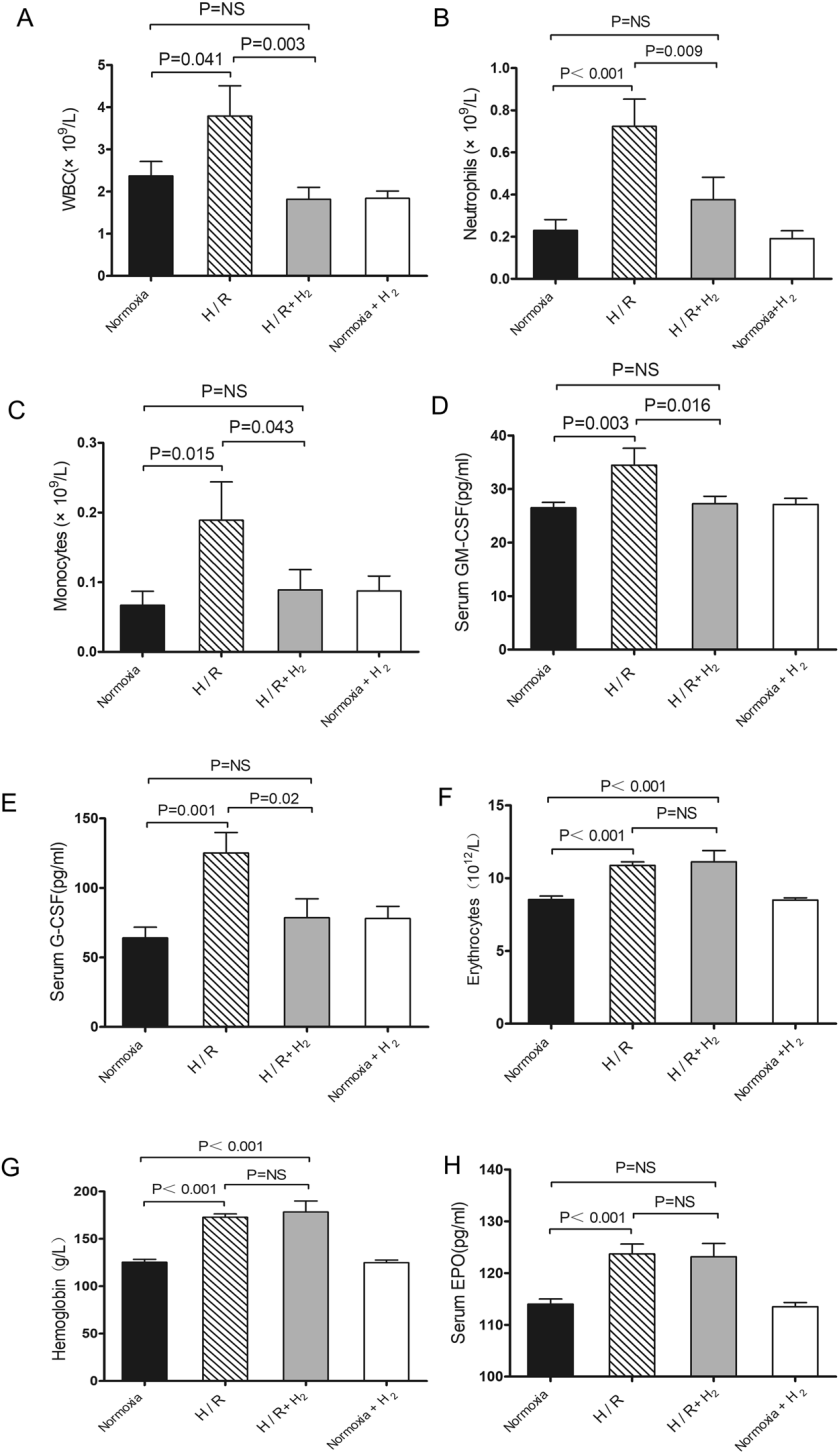
H_2_ inhibits the H/R-induced increase in G-CSF and GM-CSF, but not H/R-induced up-regulation of the EPO-erythrocyte system. Mice were treated as in Fig. 1 (n = 10 in each group). Leukocytes (**A**), neutrophils (**B**), monocytes (**C**), erythrocytes (**F**) and hemoglobin (**G**) were assayed using an automatic blood analyzer. Serum levels of G-CSF (**C**), GM-CSF (**D**), and EPO (**H**) were assayed using commercial ELISA kits. Data shown are mean ± SEM. ns, not significant.

H/R may exert its effects by up-regulation of the systems mediated by CSFs^31^ and EPO. Counts of the following cells were significantly higher in tissue from H/R-exposed animals than in tissue from normoxia-treated controls: leukocytes [(3.79 ± 0.72) × 10^9^ *vs*. (2.38 ± 0.34) × 10^9^/L, p = 0.026; Fig. 6A], neutrophils [(0.72 ± 0.13) × 10^9^ *vs*. (0.23 ± 0.05) × 10^9^/L, p < 0.001; Fig. 6B] and monocytes [(0.19 ± 0.05) × 10^9^ *vs* (0.07 ± 0.02) × 10^9^/L, p = 0.015; Fig. 6C]. H/R in the presence of H_2_ led to levels of these cells similar to those in normoxia-treated animals: leukocytes, (1.82 ± 0.28) × 10^9^/L; neutrophils, (0.38 ± 0.11) × 10^9^/L; and monocytes, (0.09 ± 0.03) × 10^9^/L (all p < 0.05 *vs*. H/R, p > 0.05 *vs*. normoxia; Fig. 6A–C).

H/R also led to significantly higher serum levels of GM-CSF than normoxia (34.46 ± 0.15 *vs*. 26.50 ± 0.98 pg/ml), as well as significantly higher levels of G-CSF (125.08 ± 14.76 *vs*. 64.03 ± 7.72 pg/ml; both p < 0.05; Fig. 6D–E). H/R in the presence of H_2_ led to levels of GM-CSF (27.26 ± 1.37 pg/ml) and G-CSF (78.57 ± 13.63 pg/ml) similar to those in normoxia-treated animals (all p < 0.05 *vs*. H/R, p > 0.05 *vs*. normoxia).

H/R led to significantly higher levels of EPO than normoxia (123.66 ± 1.90 *vs*. 114.00 ± 0.98 pg/ml), as well as significantly higher numbers of erythrocytes [(10.87 ± 0.24) × 10^12^ vs. (8.53 ± 0.24) × 10^12^/L] and hemoglobin levels (172.86 ± 3.44 *vs*. 125.27 ± 3.12 g/L; all p < 0.05; Fig. 6F–H). Levels after hypoxia in the presence of H_2_ were similar to those after H/R without H_2_: EPO, 123.15 ± 2.56 pg/ml; erythrocytes, (11.12 ± 0.78) × 10^12^/L; hemoglobin, 178.38 ± 11.63 g/L (all p > 0.05 *vs*. H/R).

These results suggest that inhaled H_2_ inhibits H/R induction of the CSF system but not of the EPO system.

### H_2_ protects CD133^+^ progenitors from H/R-induced injury

**Figure 7:**
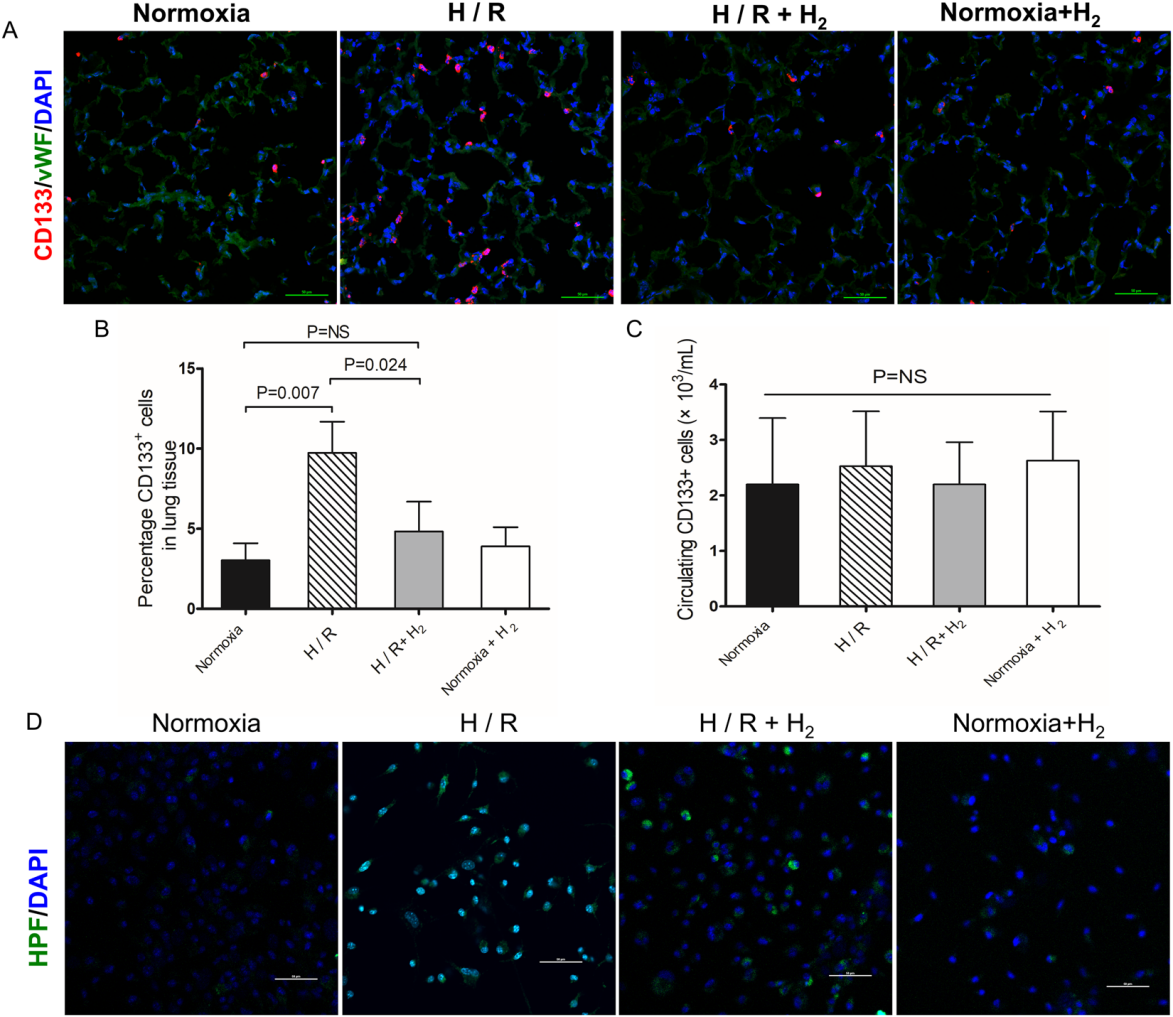
Effect of hydrogen on CD133^+^ progenitors. Mice were treated as described in Fig. 1 (n = 4 in each group). Lung samples were harvested for CD133^+^ cell staining (**A**) and counting (**B**). Blood samples were extracted and CD133^+^ cells were counted (**C**). (**D**) Cultured CD133^+^ progenitors were stained with hydroxyphenyl fluorescein solution and DAPI. Scale bar, 50 *μ*m. Data shown are mean ± SEM. ns, not significant.

Because G-CSF can mobilize progenitors from bone marrow, which home to sites of injury to mount anti-inflammatory and repair responses^32^, we wanted to test whether H/R mobilizes progenitors that home to lung tissue. Therefore, we investigated pulmonary and circulating CD133^+^ progenitors, which are pluripotential cells that can be mobilized by G-CSF^33^. These cells were abundant in lung tissues after H/R exposure, while they were rare in mice exposed to hypoxia in the presence of H_2_ and in normoxia-treated mice (Fig. 7A). Flow cytometry showed significantly higher percentages of pulmonary CD133^+^ progenitors in H/R-exposed mice (9.73 ± 1.95%) than in normoxia-treated mice (3.03 ± 1.06%, p = 0.007) and mice exposed to H/R in the presence of H_2_ (4.83 ± 1.87%, p = 0.024 *vs*. H/R, p > 0.05 *vs*. normoxia; Fig. 7B). In contrast, percentages of circulating CD133^+^ cells were similar among the various animal groups (Fig. 7C).

Next we tested the effects of H/R and H_2_ on progenitors. Cultures of mouse CD133^+^ progenitors were exposed for 8 h at 37 °C to hypoxia (10% O_2_, 5% CO_2_, 85% N_2_) or hypoxia in the presence of H_2_ (10% O_2_, 5% CO_2_, 4% H_2_, 81% N_2_). Control cultures were exposed to normoxia (5% CO_2_, 95% air) or normoxia in the presence of H_2_ (5% CO_2_, 21% O_2_, 4% H_2_, 70% N_2_). Levels of hydroxyl radicals were higher in cells exposed to hypoxia than in cells treated with normoxia, and most of the radicals colocalized with DAPI in the nucleus (Fig. 7D). H_2_ reduced the levels of hydroxyl radicals, especially those in the nucleus.

Our results suggest that H/R induces the homing of CD133^+^ progenitors to lung tissue, presumably to repair damage. H_2_ helps protect CD133 + progenitors from H/R injury by inactivating hydroxyl radicals.

## Discussion

**Figure 8:**
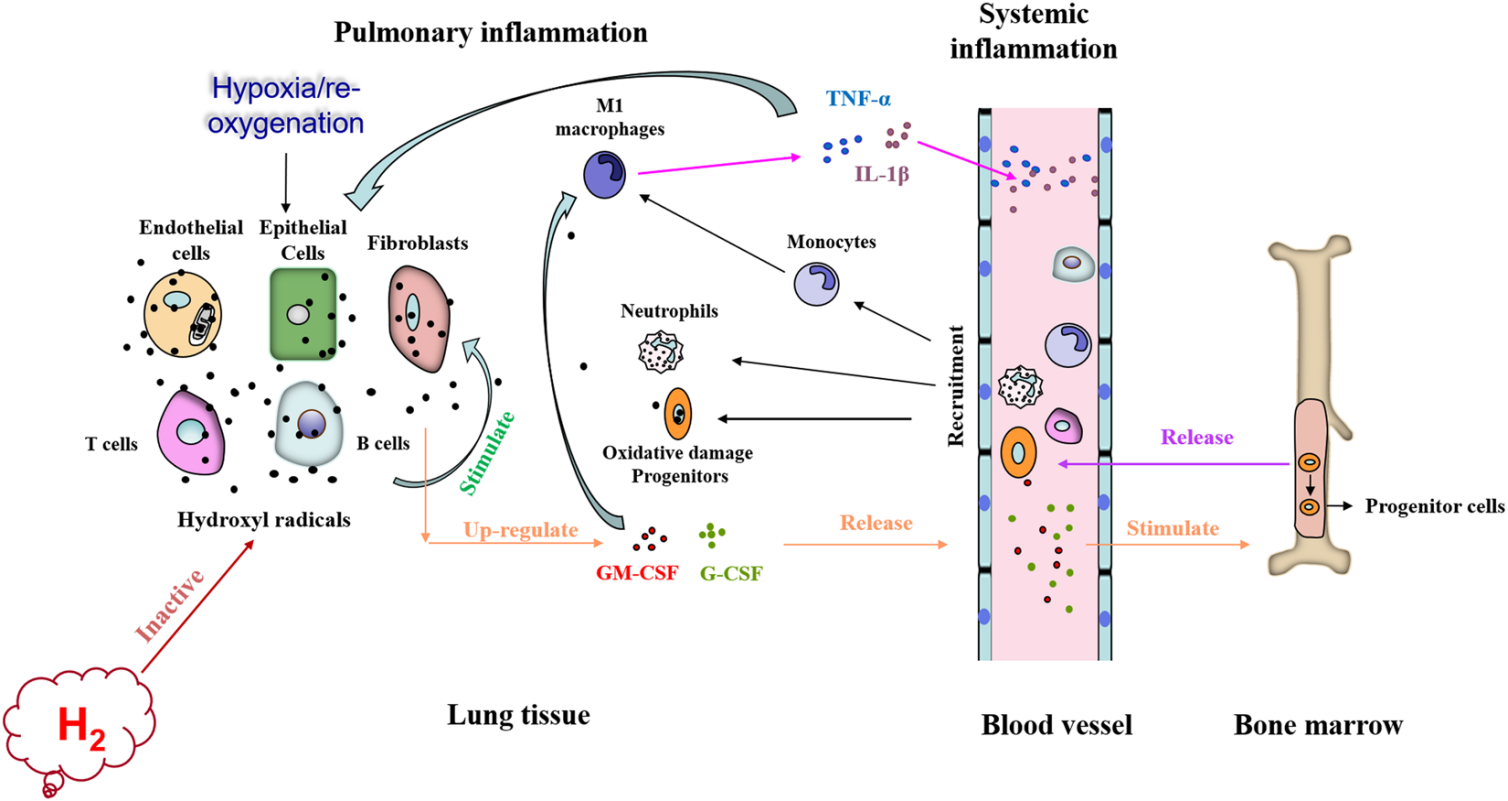
Mechanisms by which inhaled H_2_ may alleviate H/R-induced lung injury. H/R increases hydroxyl radicals, which may activate various cell types, such as epithelial cells, fibroblasts, T cells, B cells, neutrophils and macrophages. The net result is up-regulation of colony-stimulating factors, which stimulate the release of leukocytes and progenitors from bone marrow, which migrate into inflamed lung tissue. In lung tissue, infiltrated leukocytes and monocytes follow M1 polarization and release proinflammatory factors, which increase the secretion of colony-stimulating factors. The ability of infiltrated progenitors to repair lung tissue is compromised. H_2_ inactivates hydroxyl radicals induced by H/R, which down-regulates colony-stimulating factors, thereby inhibiting release of leukocytes from bone marrow and their infiltration into lung tissues. The repair ability of progenitors may also improve.

In this study, we found that mice exposed to chronic H/R exhibited significant lung injury, which was significantly improved by 4% H_2_ inhalation. H_2_ treatment inhibited the generation of hydroxyl radicals and down-regulated GM-CSF and G-CSF, which may attenuate infiltration by neutrophils and M1 macrophages, as well as release of proinflammatory factors. H_2_ may also protect the progenitor cells by inactivating hydroxyl radicals (Fig. 8). Our results demonstrate that molecular hydrogen is effective at protecting lung from H/R-induced injury.

Our results showed that H/R triggered massive generation of hydroxyl radicals, similar to previous reports^34^, and that H_2_ inhalation reduced these levels, likely reflecting the established ability of H_2_ to inactivate hydroxyl radicals^22,35^. The decrease in hydroxyl radicals was associated with reduction of GM-CSF and G-CSF, followed by down-regulation of systemic and pulmonary inflammatory responses. These results suggest that H/R-induced production of hydroxyl radicals is important for up-regulating CSFs, which can be abolished by H_2_ inhalation.

CSFs are important mediators of both systemic and tissue inflammation, but their role in H/R-induced lung injury has been unclear. GM-CSF and G-CSF can be secreted from various cell types, including epithelial cells, fibroblasts^36^, T cells, B cells^37^, neutrophils and macrophages^38^ under the stimulation of IL-1β and TNF-α^12,15^. Studies have shown that CSFs, especially GM-CSF, aggravate inflammation^39^ by enhancing proinflammatory cytokine production^40^ and mobilizing leukocytes, promoting their survival, proliferation^41^, differentiation^42^, and stimulating their activation^43–45^ and migration. Recent studies show that GM-CSF blockade has a therapeutic effect against cardiac inflammation during Kawasaki disease or aortic aneurysm formation^46^, as well as against lung diseases such as COPD^47^, interstitial lung disease^48^, allergy^49^and asthma^50^. Furthermore, GM-CSF promotes macrophage polarization toward M1-skewed cells^51^ and stimulates macrophage plasminogen activator activity^52^, which may induce up-regulation of pro-inflammatory factors. Both M1 macrophages and neutrophils may aggravate lung and systemic inflammation by releasing proinflammatory factors and elastase. In the present study, we found that H/R increased serum levels of both GM-CSF and G-CSF, which was followed by increases in leukocytes and pro-inflammatory factors in circulation and lung tissues. In addition, M1 macrophages accumulated in the lung tissues. These findings suggest that CSFs are key mediators of H/R-induced lung injury.

G-CSF not only promotes inflammation, but also mobilizes stem cells derived from bone marrow for repair^53^. To assess the role of these cells in H/R-induced lung injury, we analyzed CD133^+^ cells, a homogeneous population with multi-proliferative potential previously shown to play a role in lung repair^54,55^. We found that H/R triggered accumulation of CD133^+^ progenitors, although this did not seem to prevent lung tissue from H/R injury. In our experiments with CD133^+^ progenitor cultures, hypoxia significantly increased hydroxyl radicals. It is possible that these radicals cause oxidative injury in the progenitors, damaging their ability to repair tissue. This implies that H_2_ improves progenitor function by inactivating hydroxyl radicals in those cells. This may be another reason for the protective effects of H_2_.

In our experiments, chronic H/R increased the level of EPO in serum, which increases red blood cell count and blood viscosity, thereby aiding pulmonary artery remodeling in response to chronic H/R^56^. However, H_2_ inhalation in our mouse model of H/R-induced injury did not influence the EPO-red blood cell system, nor did it significantly affect pulmonary artery remodeling or right ventricular hypertrophy. These results suggest hydroxyl radicals activate the CSF system but not the EPO-red blood cell system.

In conclusion, our study shows that H_2_ protects lung from H/R-induced injury, at least in part by scavenging hydroxyl radicals, thereby inhibiting lung inflammation induced by G-CSF and GM-CSF. This helps protect CD133^+^ progenitors from oxidative damage. Because inhalation of 1–4% H_2_ appears to show no cytotoxicity^35^, consistent with what we found in this study, our results justify further work toward developing H_2_ as a treatment against H/R-induced lung injury, such as during acute exacerbation and remission of asthma, bronchiectasis and early COPD.

## Materials and Methods

### Animal

Male C57BL/6 mice 8 weeks old were provided by the Sichuan Provincial Experimental Animal Center. The H/R model was established as follows. Mice were placed in a closed container with inlet and outlet ports on the two sides. Sodium lime was placed at the bottom of the container to absorb CO_2_. Animals were exposed to hypoxia (10% O_2_, 90% N_2_), hypoxia in the presence of H_2_ (10% O_2_, 4% H_2_, 86% N_2_), or normoxia (21% O_2_, 79% N_2_) in the presence of H_2_ (21% O_2_, 4% H_2_, 75% N_2_) (8 h per day). Then they were exposed to air for re-oxygenation (16 h per day) for 4 weeks. Control mice were exposed to normoxia (21% O_2_, 79% N_2_) for 4 weeks. Mice had free access to water and conventional laboratory diet throughout the exposure.

Hydrogen (purity >99.9%) was generated using a hydrogen generator (Beijing Zhongxing Huili Technology Development, Beijing, China). During experiments, the O_2_ and CO_2_ concentrations were monitored using a Philips Airway Gases monitor, and H_2_ concentration was monitored using a STP1000 Multiplexed Gas Analyzer (T & P Union (Beijing), Beijing, China).

All animal experimental protocols were approved by the Ethics Committee for Animal Experiments of Sichuan University and were performed in accordance with the Guide for the Care and Use of Laboratory Animals prepared by the Institutional Animal Care and Use Committee of Sichuan University.

### Blood cell count

Blood samples were taken from the apical artery, and blood cells were counted using an automatic blood analyzer (Sysmex-XE5000, Toagosei Co., Ltd, Yokohama, Japan).

### Lung histological injury

The lung was harvested and fixed overnight in 4% paraformaldehyde at 4 °C. Paraffin-embedded sections were stained with hematoxylin and eosin, and examined under a light microscope by pathologist blinded to the experimental groups. Severity of lung injury was scored using a 5-point scale^57^ (0 = normal histology, 5 = most severe injury) that took into account the parameters of alveolar congestion, hemorrhage, neutrophil accumulation in the airspace or vessel wall, alveolar wall thickness, and hyaline membrane formation. We chose 10 images for each animal randomly, and parameter values were averaged for the 10 images. The average values of all parameters were then added together to generate a total lung injury score. Scoring was performed by two pathologists in a blinded fashion, and the two scores for a given animal were averaged to obtain the final score for that animal.

### Measurement of factors

Blood samples from the apical artery were centrifuged at 3000 rpm for 10 min at 4 °C to obtain serum. To obtain lung tissues, lung was harvested on ice immediately after euthanasia and weighed. An aliquot of tissue (50 mg) was cut into 1-mm^3^ pieces, added to 500 *μ*l PBS and homogenized. Samples were left on ice for 5 min and centrifuged at 3500 *g* for 20 min at 4 °C. Levels of EPO, G-CSF, GM-CSF, IL-1β and TNF-α in serum and lung tissue supernatant were assayed using commercial ELISA kits (Neobioscience Technology, Beijing, China).

### Evaluation of vascular remodeling and right ventricle hypertrophy

Pulmonary artery remodeling was assessed in terms of percent medial thickness, which was calculated according to the equation: (medial wall thickness × 2)/vessel diameter ×100%^58^. Only vessels with a circular appearance and external diameter between 50 and 100 *μ*m were used. Lung sections were examined by an investigator blinded to experimental treatment using an Olympus-BHS microscope.

Right ventricular hypertrophy was quantified by calculating the ratio of right ventricle to left ventricle plus septum weight [RV/(LV + S)]. The ventricles and septum were collected, and the wet and dry ventricle and septal weights were obtained by drying for 24 h at 60 °C.

### Hydroxyl radical stains and measurements

Frozen sections (5 *μ*m) of the right lung were used. Hydroxyphenyl fluorescein solution (Sigma, USA) was diluted 1:500 and added to the lung tissue section for 30 min at room temperature. Sections were washed with phosphate-buffered saline (PBS), then stained with 4′,6-diamidino-2-phenylindol (DAPI; Invitrogen, USA). Images were analyzed by confocal microscopy.

To quantify hydroxyl radicals, lung tissue (100 mg) was ground up in liquid nitrogen, added to 500 *ul* PBS and centrifuged at 10 000 g for 20 min at 4 °C. Supernatants were transferred to Eppendorf tubes and analyzed as quickly as possible using a commercial colorimetric kit (Genmed, Scientifics, USA).

### Immunofluorescence

After 4-week H/R, the right lung was harvested and cut into frozen sections (5 *μ*m), which were fixed with 4% paraformaldehyde, washed with PBS, and blocked for 1 h at room temperature with 1% bovine serum albumin (Sigma, USA) in PBS.

For detecting progenitors, rabbit anti-CD133 antibody conjugated to AF555 (Bioss, Beijing, China) and rabbit anti-Von Willebrand factor antibody conjugated to AF488 (Bioss) were used at a dilution of 1:100.

For detecting M1 macrophage in lung, sections were incubated at 4 °C overnight with rabbit anti-mouse CD86 antibody (1:100; Genetex, USA) and rat anti-mouse MOMA-2 antibody (1:50; Genetex, USA). Then the sections were washed in PBS and incubated for 1 h at room temperature with secondary antibodies conjugated to Alexa Fluor 488 or 555 (1:200; Biofroxx, Germany). As a negative control, sections were incubated with PBS instead of primary antibody. Sections were stained with DAPI (Invitrogen) to label nuclei. Images were then visualized using a fluorescence microscope (LSM 510 Meta, Carl Zeiss) equipped with a 20×/0.75D objective.

### Flow cytometric analysis of CD133^+^ progenitors

Blood was diluted 1:1 with Hank’s solution, layered onto the top of a mononuclear cell separation solution, and centrifuged at 1500 rpm for 35 min at 4 °C. The white mononuclear cell loop was obtained, washed with PBS, and incubated at 37 °C for 1 h with anti-CD133 antibody conjugated to AF555 (Bioss). Then cells were washed twice, re-suspended in 500 *μ*l PBS, and analyzed by flow cytometry on an Esp Elite device (Beckman Coulter, Chicago, IL, USA). PBS served as a negative control.

For determining pulmonary CD133^+^ cells, harvested lung tissues were placed in 2 ml PBS and 1 mg/ml Liberase^TM^ (Thermo Fisher Scientific, USA). Tissues were minced with scissors, and digested for 30 min at 37 °C before filtration through a 70-μm cell strainer and red blood cell lysis. Samples were then filtered through a 40-μm filter and resuspended, after which CD133^+^ cells were analyzed as described above.

### Cell culture

Bone marrow was collected as described^59^ by flushing the femurs and tibias of 2-month-old C57BL/6 mice with complete DMEM-LG medium (Thermo Fisher Scientific, USA). Cells were cultured for 24 h in a Petri dish (Thermo Fisher Scientific), then non-adherent cells were removed by washing with PBS. Adherent cells were further cultured in complete medium and retrieved by trypsinization with 0.25% trypsin (Thermo Fisher Scientific) for 5 min at 37 °C. Treated adherent cells were cultured and passaged three times. Third-passage CD133^+^ cells were retrieved using immunomagnetic microbeads, and further cultured in complete medium. Cultured cells were retrieved, and their morphology and ability to differentiate into osteoblasts and adipocytes were examined.

CD133^+^ cells were split into four Petri dishes (2 × 10^5^ cells/dish) and cultivated in standard medium composed of MEM alpha (20% fetal bovine serum(FBS) +1% Penicillin-Streptomycin (P/S) (Thermo Fisher Scientific) for two days, and then incubated for 8 h at 37 ◦C in a hypoxic atmosphere (10% O_2_, 5% CO_2_, 85% N_2_) or a hypoxic atmosphere containing H_2_ (10% O_2_, 5% CO_2_, 4% H_2_, 81% N_2_). Control cultures were incubated in a normoxic atmosphere (5% CO_2_, 95% air) or a normoxic atmosphere containing H_2_ (5% CO_2_, 21% O_2_, 4% H_2_, 70% N_2_). After the 8 h incubation, hydroxyphenyl fluorescein solution (1:500) and Hoechst (1:1000; Thermo Fisher Scientific) were added to cultures, which were returned to their incubators for another 30 min. Then cultures were washed with PBS, centrifuged under normoxic conditions and analyzed using confocal microscopy.

### Statistical analysis

Statistical analysis was performed using SPSS 18.0 (IBM, Chicago, USA). Results were reported as mean ± SEM. Differences between more than two groups were assessed using one-way ANOVA. The threshold for significance in all statistical tests was p < 0.05.
